# Local distribution infrastructure and robust vaccine manufacturing facilities in LMICs should be prioritised to tackle ongoing and future pandemic risk

**DOI:** 10.1016/j.lansea.2023.100158

**Published:** 2023-02-21

**Authors:** Hasan Mahmud Reza, Farhana Sultana, Razmin Bari, Jennifer Cole, Abdullah H. Baqui

**Affiliations:** a Department of Pharmaceutical Sciences, North South University, Bashundhara R/A, Dhaka, 1229, Bangladesh; b Department of Political Science and Sociology, North South University, Bashundhara R/A, Dhaka, 1229, Bangladesh; c College of Arts & Sciences, University of North Carolina at Chapel Hill, NC, USA; d Department of Health Studies, Royal Holloway University of London, Egham, Surrey, TW20 0EX, UK; e Department of International Health, Johns Hopkins Bloomberg School of Public Health, Johns Hopkins University, Baltimore, MD, 21205, USA

Vaccine hesitancy is often assumed to be the primary cause of lower vaccination rates in low-income and middle-income countries (LMICs).^1^ But when vaccines are available, the vaccine acceptance rates in LMICs are as high or higher than in high-income countries (HICs), averaging around 80%.^2^ Despite considerable literature on vaccine inequity between the Global North and Global South, the situation persists; and this warrants the key challenges to be reiterated.

## Vaccine access and distribution challenges

Despite early talks of ensuring vaccine equity during the COVID-19 pandemic, most HICs hoarded the vaccines they manufactured. The vaccination programmes were extended to prioritise the younger age groups (most vulnerable to less vulnerable) in quick succession, while large populations in LMICs were at risk from COVID-19 for a longer period.^3^ Vaccine supply was even used as a geopolitical tool^4^ and the COVID-19 death toll was four times higher in LMICs than in HICs.^5^ Vaccination equity could save a huge number of lives in LMICs but the supply of vaccines has been insufficient. For example, in Uganda, plans to fully vaccinate 20% of the population by the end of 2021 were not achieved due to unavailability of vaccines for purchase, although funds were allocated in advance.^4^^,^^6^

Lack of healthcare infrastructure has further hampered the internal distribution and access to vaccines in remote communities and attempts to motivate people to visit vaccination centres located several miles away have been met with limited success. LMICs have struggled with internal distribution challenges due to lack of ability to maintain the cold chain and appropriate storage facilities for COVID-19 vaccines^3^ just as Liberia, Sierra Leone and Guinea did for medical supplies needed during the 2014–15 Ebola crisis.^7^

## Vaccine manufacturing challenges

The HICs where most vaccines are manufactured have been reluctant to waive patent rights, which complicates knowledge sharing on technical know-how for vaccine production in LMICs. With the exceptions of larger LMICs such as India and China – where pockets of high development in urban centres mirror the HIC economy while remote rural regions remain underdeveloped – and Cuba, where healthcare has always had a special place in the political ecology, LMICs do not have significant manufacturing capacity. The Serum Institute of India suspended its commitment to international supply under COVAX when cases surged in India.^3^ As of Aug 6, 2021, China had sold nearly 1 billion doses to other countries. China had also donated 33 million to other countries, along with promises to raise this number to 100 million.^8^ Such numbers were, however, a drop in the ocean compared to the 6 billion people living in LMICs – 77% of the total world population – who require at least two doses each. Put simply, those living in LMICs do not have sufficient access to vaccines, and a large part of this challenge is the lack of in-country manufacturing capacity.

## The way forward

The solution being more complex than ‘just develop some’ is well recognised.^9^ LMICs face many health challenges, from pneumonia, malaria, HIV and Neglected Tropical Diseases (NDTs) to insufficient maternal and neonatal care, and poor sanitation. Competing priorities should not, however, side-line the vaccine challenges. To address the challenges, LMICs need international support to develop robust internal distribution systems for vaccines, especially for rural populations in remote areas. At a policy level, such programmes are often in place for childhood vaccines considered routine in HICs, such as MMR, DTP, and polio but, as with vaccines for COVID-19, it is difficult to reach remote areas due to civil war, natural disasters, or poverty.^10^ The governments of LMICs need additional budgets to strengthen its distribution infrastructures but do not have sufficient funds for the same. As the ramifications of the pandemic reverberate around the world, traditional aid donations are also diminishing. This would affect the financial assistance from international actors such as the World Bank, USAID, Gates Foundation, Warren Buffet Foundation, The Rockefeller Foundation, Wellcome Trust, as well as the technical and logistical assistance from GAVI, COVAX and WHO. In this situation, governments of LMICs will have to include support for vaccination infrastructure in their political agenda and manage funds internally. Insufficient vaccine coverage, for COVID-19 or other common diseases, leaves LMICs unable to safeguard their children, or their large, young working populations.^11^ The economic consequences of insufficient vaccine coverage are huge.^12^

One solution is for LMICs to work together to develop or expand in-country vaccine manufacturing capacity, through regional cooperation where necessary, to meet increased demand and timely supply of vaccine doses. Such action would boost their internal economies in the process. This could be achieved by identifying and strengthening capacity in LMICs, like Vietnam and Indonesia, which already have basic infrastructure and vaccine expertise. Also, countries without infrastructure could be encouraged by stressing the economic benefits of prevention strategies compared to cure.^13^ In recent years, Bangladesh has built a robust capacity for pharmaceutical manufacturing and as such could provide a case study for the successful roll-out of such a programme. India, a neighbouring country of Bangladesh, has shown the value of in-country vaccine manufacturing capacity for its own population during the COVID-19 pandemic. India could assist Bangladesh by technology transfer and provide vaccine experts at minimum cost through the South Asian Association for Regional Cooperation (SAARC). Such a programme would reduce shipment times and distribution costs, as well as provide capacity to support other vaccination programmes in Bangladesh, which is currently an under-served region of the world. GAVI and COVAX need to expand their workstreams to facilitate partnerships so that LMICs can respond to current and future pandemics. To ensure this, LMICs need to be financially stronger, which may need more large grants to be made available from international donors. The long-term value of the social, as well as financial, investment warrants such spending even when finances are constrained.^14^

## Conclusion

Enabling LMICs to achieve self-sufficiency and the agency to deliver vaccination to everyone will not be easy but is essential if we are to achieve a COVID-resilient world. HICs need to be more supportive to LMICs directly, and indirectly through GAVI or COVAX, by providing financial, technological, and operational assistance to build in-country capacity. Such investment is essential to the prevention of global economic losses from vaccine-preventable disease.^10^^,^^12^ We appeal to international funding agencies to consider this as the way forward from the inequity and unequal impact of the COVID-19 pandemic. We acknowledge that this appeal has been made before but, it needs to be repeated until it is heeded.

## Contributors

HMR - conceptualisation, formal analysis, project administration, supervision, writing - original draft, and writing - review and editing. FS - literature search, data formal analysis, and writing - original draft. RB - literature search and writing - original draft. JC - literature search and writing - original draft, and writing - review and editing. AB - critical reading and editing.

## Declaration of interests

None.
